# Comparison of flow characteristics and vascular reactivity of radial artery and long saphenous vein grafts [NCT00139399]

**DOI:** 10.1186/1749-8090-1-4

**Published:** 2006-03-03

**Authors:** William CF Chong, Peter Collins, Carolyn M Webb, Anthony C De Souza, John R Pepper, Christopher S Hayward, Neil E Moat

**Affiliations:** 1 Department of Cardiothoracic Surgery, Royal Brompton and Harefield NHS Trust, London, UK; 2 Department of Cardiology, Royal Brompton & Harefield NHS Trust, London, UK; 3 Cardiac Medicine, National Heart and Lung Institute, Imperial College London, London, UK; 4 Department of Cardiology, St Vincent's Hospital and Victor Chang Cardiac Research Institute, Sydney, Australia

## Abstract

**Background:**

The morphological and functional differences between arteries and veins may have implications on coronary artery bypass graft (CABG) survival. Although subjective differences have been observed between radial artery (RA) and long saphenous venous (LSV) grafts, these have not been quantified. This study assessed and compared the flow characteristics and *in-vivo *graft flow responses of RA and LSV aorto-coronary grafts.

**Methods:**

Angiograms from 52 males taken 3.7 ± 1.0 months after CABG surgery were analyzed using adjusted Thrombolysis in Myocardial Infarction (TIMI) frame count. Graft and target coronary artery dimensions were measured using quantitative coronary angiography. Estimated TIMI velocity (V_E_) and volume flow (F_E_) were then calculated. A further 7 patients underwent *in-vivo *graft flow responses assessments to adenosine, acetylcholine and isosorbide dinitrate (ISDN) using intravascular Doppler.

**Results:**

The V_E _for RA grafts was significantly greater than LSV grafts (*P *= 0.002), however there was no difference in volume F_E _(*P *= 0.20). RA grafts showed positive endothelium-dependent and -independent vasodilatation, and LSV grafts showed no statistically significant response to adenosine and acetylcholine. There was no difference in flow velocity or volume responses. Seven RA grafts (11%) had compromised patency (4 (6%) ≥ 50% stenosis in the proximal/distal anastomoses, and 3 (5%) diffuse narrowing). Thirty-seven (95%) LSV grafts achieved perfect patency and 2 (5%) were occluded.

**Conclusion:**

The flow characteristics and flow responses of the RA graft suggest that it is a more physiological conduit than the LSV graft. The clinical relevance of the balance between imperfect patency versus the more physiological vascular function in the RA graft may be revealed by the 5-year angiographic follow-up of this trial.

## Ultramini abstract

We compared 3-month post-operative flow characteristics and *in-vivo *flow responses of radial artery (RA) and long saphenous vein (LSV) grafts. Basal velocity was significantly greater in RA versus LSV grafts, but volume flow was similar. RA grafts, but not LSV grafts, showed endothelium-dependent vasodilatation.

Interest in the radial artery (RA) as a coronary artery bypass graft has grown over recent years, driven by a desire to replace vein grafts with arterial grafts. Numerous observational series have shown favourable early and longer-term patency results, summarized in a recent review [1]. Furthermore, the RA has been used successfully as a composite conduit [2]. One mid-term angiographic review in patients attending for angiography due to post-operative symptoms found decreased patency in RA grafts versus left internal mammary artery (LIMA) or long saphenous vein (LSV) grafts, particularly in women, suggesting a potential need for selectivity in RA graft use [3]. A recent multicentre randomized controlled study demonstrated lower graft occlusion rate in RA versus LSV grafts to either the left circumflex (LCx) or right coronary artery (RCA) at 10 month angiographic follow-up [4]. At 5 year follow-up, a prospective, randomized, single centre trial reported no difference in patency between RA and free right internal thoracic artery or LSV when grafted to a region at the surgeon's discretion, however only 30% of patients returned for angiography [5]. To date there is no randomized trial to compare the RA with SVG grafts to a single coronary territory.

The success of arterial grafts has been attributed to more physiological flow characteristics and adaptability to the coronary arterial bed by a process of autoregulation [6]. LSV grafts are generally larger than native coronary arteries and, together with the presence of valves, may have flow characteristics that are very different from those of the native coronary arteries. LSV graft flow pattern has been shown to be different from pedicled left internal mammary artery (LIMA) grafts [7], and LIMA grafts have similar endothelium-dependent and -independent responses to RA grafts early (3 weeks and 6 months) post-operatively [8]. However, the flow characteristics and the *in-vivo *flow responses in RA grafts have not been compared with LSV grafts. The aim of this study therefore was to examine the resting flow characteristics and dynamic flow responses in RA and LSV aorto-coronary bypass grafts to a single coronary artery territory.

## Methods

### Patients

Patients undergoing myocardial revascularization surgery aged 40–70 years with significant stenosis (≥ 70%) in the circumflex territory as identified on preoperative angiograms, and a negative Allen's test (defined as the return of palmar circulation within 5 seconds of releasing ulnar artery compression) were enrolled. Exclusion criteria were poor LV function (EF <25%), severe diffuse peripheral vascular disease or bilateral varicose venous disease and inability to comply with the angiographic follow-up at 3 months or/and 5 years.

Ethics approvals for both the randomized study and the *in vivo *graft flow sub-study were obtained from the Royal Brompton Hospital Ethics Committee. Written informed consent was obtained from all patients preoperatively.

### Study design

The Radial artery versus Saphenous Vein Patency (RSVP) trial is a prospective randomized trial to compare angiographic patency rates of RA and LSV grafts to the native LCx territory. Phase 1, the results of which are presented here, was designed as a safety/efficacy assessment to address any early technical problems associated with using RA to the ascending aorta and involved the first 100 patients returning for 3 month post-operative angiography. A subgroup of these patients also underwent physiological assessment of the randomized graft at angiography (described below). Randomization was 2:1 in favour of RA to increase the RA graft dataset. Phase 2 involved recruitment of additional patients, and patients from phase 1 and 2 will undergo assessment of long-term angiographic graft patency at 5 years.

### Angiography and analysis

All vasoactive medications such as β-blockers, calcium antagonists, nitrates, angiotensin converting enzyme inhibitors and statins were stopped 24 hours prior to study. Angiograms were performed using 5 or 7 French angiographic catheters with Omnipaque contrast medium. Frame acquisition was 25 frames/s. Heart rate and blood pressure were recorded throughout the study. Angiographic endpoint was graft patency graded to the following scale: P1 (perfect patency, no irregularities), P2 (<50% stenosis – proximal or distal anastomoses or body; multiple or single), P3 (≥ 50% stenosis – proximal or distal anastomoses or body; multiple or single), P4 (diffuse narrowing, 'string sign') or P5 (total occlusion). All stenoses were assessed using quantitative coronary angiography (QCA; Medis, NL). Graft diameter and length and target coronary artery diameter were also analyzed using QCA as previously described [9]. Analysis was performed by an independent investigator.

### TIMI flow characteristics

Flow characteristics in both grafts were assessed using the TIMI frame count. This is a reproducible and semi-quantitative measure of coronary flow index represented as time in seconds (TIMI frame count/25 frames = seconds) [10] and previously has been applied to LSV grafts [11]. Total number of frames were counted from the initial complete opacification of the proximal anastomosis of the graft to the frame where dye first enters the native coronary at the distal anastomosis [12]. TIMI flow velocity estimate (V_E_) was derived using the following equation [12]:



Flow volume estimate (F_E_) (ml/s) was calculated by multiplying V_E _with cross-sectional area [12]. TIMI analysis was conducted by independent investigators.

Intra-observer and inter-observer variability of TIMI frame count was assessed in angiograms from 10 patients. Correlation coefficients were r = 0.96 and 0.92 respectively for repeated measurements.

### Pharmacological challenge

In a subgroup of patients, endothelium-dependent and -independent graft function was assessed. After confirmation of patency of both right coronary artery (RCA) and LCx grafts, patients were heparinized and a 0.014 inch Doppler wire (Cardiometrics@ Inc, Mountain View, California) was positioned in the proximal third of the randomized obtuse marginal or RCA grafts. A continuous trace of average peak blood flow velocity was recorded together with arterial blood pressure, heart rate and ECG.

After equilibration, an intra-graft bolus of the endothelium-independent vasodilator adenosine (30 μg) was given via the guiding catheter, followed by two 2-minute infusions of the endothelium-dependent vasodilator acetylcholine 10^-7 ^and 10^-6 ^M. The Doppler wire was then withdrawn and inserted into the second graft (either the LCx or RCA graft) and the above infusions repeated. Finally an intra-graft bolus of ISDN (300 μg) was given. The Doppler wire was then re-introduced into the first graft and the ISDN infusion repeated.

Velocity was measured at peak velocity response or 2 minutes after commencement of infusion. Angiograms were performed at baseline and at peak velocity response to each vasoactive substance. There was a rest period of at least 1 minute between each infusion to allow all measured parameters to return to baseline.

### Statistics

Nominal data and patency rates between the two grafts were analyzed using Chi square test for proportions. Continuous variables were analyzed using unpaired t-tests with significance at p < 0.05 and are expressed as mean ± SEM. TIMI flow data were analyzed using unpaired Student's t test and *in-vivo *graft data were analyzed as a within patient comparison using a paired Student's t test. Because of the difference in territory compared for the *in-vivo *graft data, the percentage dilatation achieved was divided by the grafted coronary artery diameter response to isosorbide dinitrate. Significance was set at 5%. Data are expressed as mean ± SEM.

## Results

### Patients

One hundred patients underwent early angiographic graft patency assessment at mean of 3.7 ± 1.1 months after surgery. Sixty-one patients were randomized to RA grafts (aged 58.7 ± 0.8 years) and 39 to LSV grafts (aged 59.4 ± 1.3 years), with no difference in patient characteristics between groups (1 female in each group, diabetes 8 vs 20%, hypertension 59 vs 51%, hypercholesterolemia 77 vs 82%, smoking history 79 vs 72%, previous MI 49 vs 59%, RA vs LSV graft patients respectively; all p = NS). Operative and post-operative details such as bypass and cross-clamp times, intensive care stay and duration of hospital stay were not statistically different.

### Graft patency

All RA grafts were patent with no incidence of complete occlusive disease. Fifty-four (89%) patients had RA grafts that were perfectly patent (grade P1), however 7 patients (11%) had compromised patency with 4 (6%) in grade P3 (≥ 50% stenosis in the proximal/distal anastomoses), and 3 (5%) in grade P4 (diffuse narrowing typical of string-sign). Thirty-seven (95%) LSV grafts achieved grade P1 and 2 (5%) were completely occluded (grade P5). There were no intermediate grades seen in the LSV graft group. There was no significant difference between the two groups in each of the grades.

Intra-graft nitrate was infused down all compromised RA grafts. This did not affect the degree of anastomotic narrowing of RA grafts in grade P3 but all of the RA grafts with string-sign exhibited a small degree of dilatation. Grade P3 RA grafts all had good rapid flow of angiographic contrast through the grafts, despite significant anastomotic narrowings (>75%). All patients with occluded or compromised grafts were clinically well and symptom free. There were no major adverse events related to graft occlusion or graft stenosis.

### Graft dimensions

**Figure 1:**
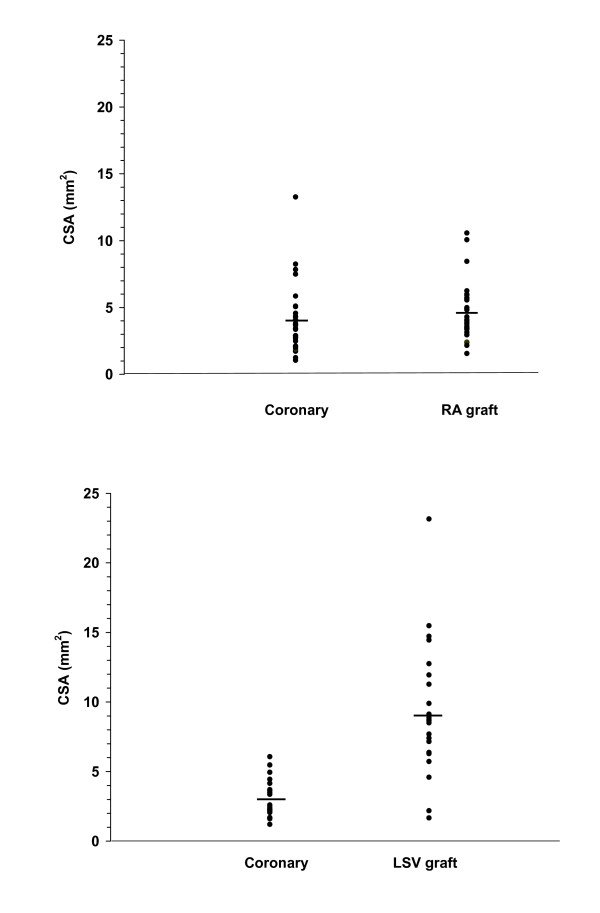
Cross-sectional areas of target coronary artery versus respective radial artery graft (top) or long saphenous vein graft (bottom).

RA graft diameters were significantly smaller than LSV grafts (2.29 ± 0.09 vs 3.23 ± 0.13 mm respectively, P < 0.001), and graft-coronary diameter difference (0.19 ± 0.07 vs 1.34 ± 0.13 mm) and cross-sectional area difference were significantly smaller for RA grafts than LSV grafts (both P < 0.0001; Figure 1) indicating better size-matches for RA to their target coronary arteries. There was no significant difference in the target coronary artery diameter (P = 0.16) and graft length (10.13 ± 0.49 vs 9.35 ± 0.46 cm, P = 0.29) between graft types.

### TIMI flow characteristics

Angiograms from 52 patients were suitable for analysis of TIMI flow characteristics. There was no difference in patient characteristics between groups (30 vs 22 patients, aged 60 ± 1 vs 60 ± 2 years, 3.3 ± 0.1 vs 3.3 ± 0.2 grafts, RA vs LSV graft respectively; all p = NS). Others were excluded from analysis due to a variety of technical considerations such as incomplete opacification of the whole graft. Mean V_E _for RA grafts was significantly greater than for LSV grafts (18.35 ± 1.4 vs 11.86 ± 1.17 cm/s, *P *= 0.002), however the mean volume F_E _was not significantly different (47 ± 5 vs 58 ± 6 ml/min, *P *= 0.20). Heart rate and mean ascending aortic pressure during angiography were not significantly different between the 2 groups (75 ± 3 vs 73 ± 2 bpm and 114 ± 3 vs 112 ± 3 mmHg, RA vs LSV graft and HR vs MAP respectively).

### Pharmacological challenge

**Figure 2:**
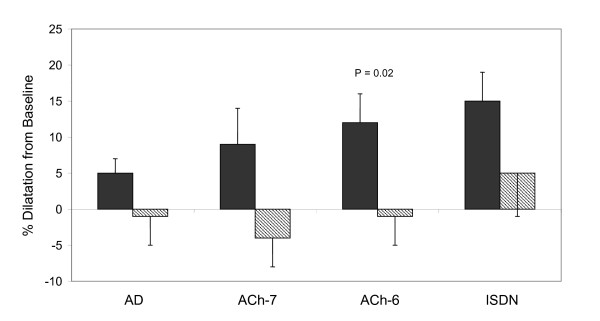
Graft vasodilatation to pharmacologic stimulation in RA (dark bars) and LSV (hatched bars) grafts after correction for native coronary artery territory.

Seven patients were included in the sub-study (aged 57 ± 4 years). Mean vascular reactivity data of RA and LSV grafts in response to pharmacological challenge are presented in Table 1. RA grafts increased diameter in response to adenosine, acetylcholine and ISDN compared to baseline. In contrast, LSV graft diameters did not exhibit statistically significant change in response to adenosine, acetylcholine or nitrate. After correcting for differences in diameter responses in the grafted territory, there was a significant percentage index diameter increase in RA versus LSV grafts when exposed to ACh 10^-6 ^mmol (Figure 2).

**Table 1:** Mean data for pharmacological challenge study

Intervention	Diameter (mm)		Velocity (cm/s)		Flow (ml/min)		Resistance (mmHg/ml/min)	
	RA	LSV	RA	LSV	RA	LSV	RA	LSV
Baseline	2.5 ± 0.04	3.51 ± 0.17	19.1 ± 2.7	14.5 ± 1.3	30.2 ± 2.3	48.4 ± 5.4	4.5 ± 0.5	3.6 ± 0.5
Adenosine	2.69 ± 0.06*	3.56 ± 0.16	43.7 ± 4.6**	39.4 ± 3.2***	81.2 ± 6.6*	123.4 ± 10.2*	1.8 ± 0.2*	1 ± 0.1*
ACh 10-7M	2.79 ± 0.04*	3.47 ± 0.14	21.9 ± 2.9	17.9 ± 2.4	42.5 ± 3.4*	51.4 ± 4.6	3.7 ± 0.5	3.1 ± 0.5
ACh 10-6M	2.78 ± 0.06*	3.57 ± 0.16	26.7 ± 4.2	22.8 ± 3.48	52.2 ± 4.9	66.2 ± 6.8	3 ± 0.3	2.3 ± 0.3
ISDN	2.84 ± 0.05*	3.63 ± 0.18	30.8 ± 4.8	29.6 ± 2.5***	63.2 ± 6.2*	93 ± 6.8*	3.6 ± 0.6	1.3 ± 0.1*

Values are mean ± SEM. *P ≤ 0.05, **P ≤ 0.01, ***P ≤ 0.001 compared to baseline.

ACh = acetylcholine.

While velocity percent changes from baseline were slightly higher in LSV grafts, and slightly higher in RA grafts with respect to flow, there was no statistically significant differences in velocity or flow responses between RA and LSV grafts to any intervention. Adenosine induced significant increases from baseline in blood flow velocity and volume flow in both arterial and venous grafts, and both significantly increased flow from baseline in response to nitrate. Interestingly, there was a significant flow increase from baseline to acetylcholine 10^-7 ^M in RA grafts (P = 0.05), and a trend at acetylcholine 10^-6 ^M (P = 0.08) but no such trend in LSV grafts (both P = 0.7 and 0.1 respectively). Resistance decreased significantly from baseline in response to adenosine in both arterial and venous grafts, and to nitrate in LSV grafts, but there was no significant difference between graft types. There was no difference in coronary flow reserve for the two graft types (2.9 ± 0.2 vs 3 ± 0.2, RA vs LSV respectively; P = 0.8). Heart rate and mean blood pressure did not change significantly throughout the procedure (75 ± 14 bpm and 114 ± 17 mmHg respectively).

## Discussion

This single-centre randomized study compares the patency, early flow characteristics and flow responses to pharmacological stimulation of flow in arterial and venous grafts. Prospectively randomizing the patient to receive RA or LSV grafts and controlling for disparities in native coronary anatomy by assigning the randomized graft to a single (the LCx) territory, ensures meaningful clinical comparisons can be made. We showed that RA grafts are better matched in both structure and function to the native coronary artery than LSV grafts. TIMI velocities were greater but flows were no different in RA versus LSV grafts consistent with adequate revascularization at rest. Pharmacological challenge revealed preserved endothelium-dependent vasodilatation in RA but not LSV grafts 3 months post-CABG surgery.

### Comparison of graft patency

Early RA graft patency rates were comparable to LSV graft patency, in line with published case series [1]. There were no early RA graft occlusions but 11% had compromised function such as anastomotic narrowing or diffuse narrowing indicated by the 'string-sign'. Proximal anastomotic problems with the RA all occurred early in the trial and were a feature of surgeons with less experience with the radial arterial conduit, and may reflect a learning curve. This problem was not seen later in the trial. Despite the presence of fixed anastomotic stenoses in some RA grafts, they were still patent with good antegrade flow in the remainder of the graft, whereas LSV grafts were either perfectly patent or totally occluded.

### Relative diameters of native coronary artery and grafts

There was a much better size match to the target LCx with the RA. It is possible (but very unlikely) that the size of an individual's RA is approximately the same as the largest branch of the LCx coronary artery. However, it is likely that the RA graft, with its preserved endothelial function, is capable of autoregulating its size to adapt to the target coronary circulation, as was reported with the pedicled left internal mammary artery graft [6].

The haemodynamic effects of the internal diameter disparity between native coronary arteries and respective vein grafts has not been well studied. Stoel and colleagues calculated flow in the native LCx of 67 ml/min after giving intracoronary nitrate, which is similar to our nitrate-induced RA flow (63 ml/min) but quite different from the LSV flow (93 ml/min) in our patients [13]. This raises the possibility of turbulence and marked change in flow pattern at the site of distal anastomosis with LSV grafts which may have implications on long-term anastomotic patency.

### Graft diameter responses to pharmacological challenge

Intra-graft infusions of adenosine and acetylcholine resulted in divergent effects on graft diameters; RA grafts dilated to both drugs whereas there was a slight but statistically non-significant diameter decrease to adenosine and acetylcholine in LSV grafts. After adjusting for disparities in native coronary artery studied, this difference was statistically significant at the higher dose of acetylcholine. A dilatory response to acetylcholine indicates a functioning endothelium in RA grafts at 3 months after surgery, as previously reported 5 years post-operatively [14]. The inability of LSV grafts to show endothelium-dependent vasodilatation indicates that endothelial dysfunction is present as early as 3 months after surgery, similar to a study of chronic LSV aorto-coronary grafts [15]. This early endothelial injury may be the result of surgical preparation of LSV conduits through excessive distension [16,17], although in our study both RA and LSV grafts were subjected to the same distension pressure [18], and may be further propagated by cyclical systemic pressure distension in situ. In the long-term this may lead to changes in gene expression and ultimately to vein graft adaptation and intimal hyperplasia/atherosclerosis, with narrowing of luminal area subsequently involving the media [19]. Such injury is probably not seen in RA grafts, as shown by our in-vitro laboratory studies [18]. Chronic LSV aorto-coronary grafts have been reported to have minimal vasodilatory reserve to nitrates [15], however a vasodilatory response to nitrate in our study confirmed a functioning smooth muscle media in the LSV grafts, indicating that the injury 3 months post-operatively is localized to the endothelium.

### Flow dynamics of arterial and vein grafts

RA graft TIMI flow velocities were significantly greater than LSV grafts, similar to previously published data [11,12], although direct measurement using intravascular Doppler revealed a smaller difference. This may be said to be predictable given the same total volume flow down the smaller diameter of the RA grafts, however it may be of clinical significance. Larger flow velocities are associated with greater shear stress on the vessel wall. Shear stress is known to induce compensatory mechanisms in endothelial cells such as inducing local vasodilator release, including nitric oxide and prostaglandins, and inhibiting constrictor factors such as endothelin [20-22], and possibly beneficially affecting neutrophil adhesion [23] and smooth muscle cell proliferation [24]. LSV grafts, without evidence of an intact endothelium, may be subjected to the adverse effects of wall shear stress changes associated with alteration in flow demands and the vessel wall may be subjected to excessively high shear stress which may led to further injury [25,26]. It has been suggested that the intimal hyperplasia commonly seen in LSV grafts may in fact represent an attempt to normalize wall shear stress by vascular remodeling [27].

TIMI-derived volume flow was not statistically significantly different between graft types, perhaps reflecting the similar target territory (LCx coronary artery) and distal run-offs, but were comparable to those measured previously using intra-operative volume flowmetry [28]. Although we did not directly measure flow in the native coronary artery beyond the graft, given the similar target territory and the comparable diameters of the RA grafts and native coronary arteries, we suggest that native arterial flow patterns would be more similar to RA grafts than LSV grafts. In a previous study LCx nitrate-induced flow was 67 ml/min, comparable to our RA graft measurements but not the LSV measurements [13]. The implication is that there would be a fairly major transition in the flow physiology at or around the distal anastomosis between LSV grafts and the native coronary artery. Turbulent flow may predilect to accelerated atherosclerotic formation [27].

Percentage increases in volume flow from baseline in response to pharmacological agents were similar in both groups indicating that RA grafts, despite their smaller size, are capable of delivering a similar volume of blood as the LSV grafts to meet myocardial oxygen demand. As endothelial function is preserved in RA grafts, we propose that they provide a more physiological autoregulation of flow compared with LSV grafts.

### Limitations

The limitations of the TIMI frame count for estimating flow and velocity have previously been discussed [11]. Variables such as heart rate and blood pressure, which may affect TIMI frame count, were not significantly different between groups. Variability resulting from injection rate of angiographic contrast media has been shown to be small and insignificant [29]. Catheter size (5 vs 7 French) does not appear to affect TIMI frame count (p = 0.2).

Length of both grafts was measured with quantitative coronary angiography and this may vary according to geometry and spatial position of the grafts and cardiac cycle, which may contribute to some degree of error in the measurement obtained. To overcome this, only the longest measurement of length of the graft in the left anterior oblique view at diastole was taken. Another limitation was that native coronary flow velocity was not estimated nor directly measured in this study and the data used in the discussion were referenced from other studies [12].

Due to the complexity of the protocol we were only able to enroll a small number of patients into the pharmacological challenge part of the study. It is possible that some of the trends shown may have shown significance if larger numbers were able to be included.

## Conclusion

The 3 month follow-up of the RSVP trial showed confirms excellent, although not perfect, early patency of RA grafts as compared to those of LSV grafts. There were no occluded RA grafts in the present series and an absence of any significant post-operative complications arising from using the RA as a coronary conduit. Our findings suggest that the RA is a more physiological conduit than the LSV, exhibiting flow characteristics that are comparable to the native coronary system with intact endothelial function 3 months after CABG surgery. The clinical relevance of the balance between imperfect patency versus the more physiological vascular function in the RA graft may be revealed by the 5-year angiographic follow-up of this trial.
